# Impact of traumatic dental injuries and malocclusions on quality of life of young children

**DOI:** 10.1186/1477-7525-9-78

**Published:** 2011-09-24

**Authors:** Janaina M Aldrigui, Jenny Abanto, Thiago S Carvalho, Fausto M Mendes, Marcia T Wanderley, Marcelo Bönecker, Daniela P Raggio

**Affiliations:** 1 Department of Pediatric Dentistry and Orthodontics, Dental School, University of São Paulo-USP, São Paulo, Brazil

**Keywords:** tooth injuries, malocclusion, oral health-related quality of life, preschool child

## Abstract

**Background:**

The presence of traumatic dental injuries and malocclusions can have a negative impact on quality of life of young children and their parents, affecting their oral health and well-being. The aim of this study was to assess the impact of traumatic dental injuries and anterior malocclusion traits on the Oral Health-Related Quality of Life (OHRQoL) of children between 2 and 5 years-old.

**Methods:**

Parents of 260 children answered the six domains of the Early Childhood Oral Health Impact Scale (ECOHIS) on their perception of the OHRQoL (outcome). Two calibrated dentists assessed the types of traumatic dental injuries (Kappa = 0.9) and the presence of anterior malocclusion traits (Kappa = 1.0). OHRQoL was measured using the ECOHIS. Poisson regression was used to associate the type of traumatic dental injury and the presence of anterior malocclusion traits to the outcome.

**Results:**

The presence of anterior malocclusion traits did not show a negative impact on the overall OHRQoL mean or in each domain. Only complicated traumatic dental injuries showed a negative impact on the *symptoms *(p = 0.005), *psychological *(p = 0.029), *self image/social interaction *(p = 0.004) and *family function *(p = 0.018) domains and on the overall OHRQoL mean score (p = 0.002). The presence of complicated traumatic dental injuries showed an increased negative impact on the children's quality of life (RR = 1.89; 95% CI = 1.36, 2.63; p < 0.001).

**Conclusions:**

Complicated traumatic dental injuries have a negative impact on the OHRQoL of preschool children and their parents, but anterior malocclusion traits do not.

## Introduction

Traumatic Dental Injury (TDI) is a common oral disorder in preschool children, since, during this period, the young child is learning to crawl, stand, walk and run. The rudimentary stage of development of reflexes and the lack of motor coordination may lead to falls. These are the principal cause of TDI in this population [1-4]. In Brazil, the prevalence of TDI ranges from 9.4% to 41.6% [4-7]. This variation may be caused by the differences in methods of data collection, sample selection or place where study was conducted [6].

Traumatic injury is a distressing experience on physical level, but it may also have an effect on emotional and psychological levels [8]. Moreover, TDI may result in pain, loss of function, and it could adversely affect the developing occlusion and aesthetics. These situations could have a negative impact on these children lives.

Upper central incisors are the teeth more frequently affected by trauma, possibly because of their position in mouth, being less protected than other teeth [5,9,10]. The presence of an increased incisal overjet and anterior open bite are physical features that have been reported as predisposing factors of TDI [5,11-14]. Moreover, the presence of these anterior malocclusions traits (AMT) may cause loss of function and aesthetics problems by themselves.

The concept of Oral Health-Related Quality of Life (OHRQoL) corresponds to the impact which oral health or diseases have on the individual's daily functioning, well-being or overall quality of life. Oral diseases and disorders during childhood can have a negative impact on the life of preschool children, affecting their growth, weight, socializing, self-esteem, and learning abilities, and also on the quality of life of their parents [15-17]. Studies have developed and tested different OHRQoL questionnaires for children aged from 6 years or older [18-22]. For younger children, on the other hand, this kind of research is limited, and the Early Childhood Oral Health Impact Scale (ECOHIS) [16] was developed to assess the burden of dental diseases and its treatment among young children in epidemiological surveys. To capture the child's entire lifetime's experience, it uses response options that assess the frequency in which oral diseases and treatments affect a child's OHRQoL. It has been also translated to Brazilian Portuguese [23], but up to this date, TDI and AMT, specifically, have not been tested yet in relation to OHRQoL in this age group.

As TDI and AMT may affect the children physically, emotionally and psychologically, and due to the lack of researches testing OHRQoL in young children, the purpose of this study was to assess the impact of TDI and AMT on the OHRQoL of preschool children and their parents.

## Material and methods

This study was reviewed and approved by an independent ethical board, Faculty of Dentistry, at Universidade de São Paulo, protocol number 36/2009.

### Study population and data collection

For this cross-sectional study, preschool children aged from 2 to 5 years of both genders and their parents, who sought dental care during the screening program of the Dental School, University of São Paulo, were asked to participate in the study (population size N = 305). The screening program is free and open to the whole population aged 0 to 7 years in the city who wants dental treatment. The inclusion criteria for the study were: children not undergoing orthodontic treatment, with parents fluent in Brazilian Portuguese and who were willing to participate in the study. This study was carried out with a total of 260 parents and children who agreed to participate in the research by a parents' authorization in a signed consent form (positive response rate of 85.2%).

On the day of the dental screening, one of the parents was invited to answer a questionnaire on children's OHRQoL (ECOHIS). The interviews were carried out by two interviewers blinded to oral examinations. They were trained in the reading and intonation of each question and option of responses. The child's oral examination for Early Childhood Caries (ECC), TDI and AMT was independently carried out by two calibrated examiners. The inter-examiner reliability was established by re-examination of 26 (10% of sample) children and they obtained values of Kappa agreement of 0.8 for ECC, 0.9 for TDI and 1.0 for AMT. Advices and comments to parents, about their children's oral health, were only given after they had answered the OHRQoL questionnaire, in order not to influence their responses.

### Children's' oral examination

The examinations for TDI, AMT and ECC were performed in a dental unit using an operating light, a 3-in-1 syringe, tongue depressors and periodontal probes.

Types of TDI were classified in clinical examination, according to Glendor et al., 1996 [24]. Uncomplicated injuries were defined as those in which the pulpal tissue was not exposed and the tooth was not dislocated (crown fracture of enamel only, crown fracture of enamel and dentin, concussion, subluxation). Complicated injuries involved exposure of the pulpal tissue and/or dislocation of the tooth (complicated crown fracture, root fracture, lateral luxation, extrusive luxation, intrusive luxation and avulsion).

Besides this classification, the presence of crown discoloration was also assessed. This feature is common sequelae of TDI and causes aesthetics problems. The discoloration could be yellow, pink, brown or grey. The authors considered teeth with crown discoloration as probably having suffered a concussion or a subluxation, therefore these teeth were classified as uncomplicated injuries.

The unit of analysis was the individual child. The child was considered as having TDI when at least one kind of trauma was present; otherwise the child was considered with absence of TDI (tooth present and sound). The presence of at least one tooth with complicated TDI classified the child with complicated trauma.

The AMTs assessed were: Anterior Open Bite - lack of a normal superposition in any of the anterior incisors - and Overjet - the horizontal distance between the incisal edges of upper and lower central incisors greater than or equal to 3.1 mm [25-27]. The presence of at least one of these AMT classified the child as having AMT, otherwise, the child was considered as not having AMT.

ECC was assessed according to the World Health Organization criteria (WHO) [28] and calculated in terms of decayed, indicated for extraction and filled primary teeth (dmf-t). The dmf-t was categorized according to the severity of ECC, and children were individually classified based on the previously proposed scores [29]: dmf-t 0 = caries free; dmf-t 1-5 = low severity; or dmf-t ≥6 = high severity.

The data on caries were used in this paper to adjust the results in the regression analyses. The effect of caries on OHRQoL in preschool children has been thoroughly addressed in another paper [30].

### Early Childhood Oral Health Impact Scale (ECOHIS)

The Brazilian version of ECOHIS [23] was used to assess the children's oral health related quality of life. It considers the child's entire lifetime's experience of dental disease and treatment in parent's responses. The Brazilian version of ECOHIS evaluates the perception of parents on OHRQoL of their 2- to 5-year-old children. It contains 13 questions corresponding to six domains, where four are on the child impact section: *symptoms *- 01 item; *function *- 04 items; *psychological *- 02 items; *self-image/social interaction *- 02 items; and two domains are on the family impact section: *parent distress *- 02 items and *family function *- 02 items. Response categories for the ECOHIS are coded: 0 = never; 1 = hardly ever; 2 = occasionally; 3 = often; 4 = very often; 5 = don't know. The total ECOHIS scores, and scores for individual domains, were calculated as a simple sum of the response codes. The number of "I don't know" responses was counted, but they were excluded from the total ECOHIS score for each patient. Questionnaires having two or more unanswered items in the domains related to the child, or one or more unanswered item in the domains related to the family, were excluded from the analysis. Higher scores indicate a more negative impact on the OHRQoL or vice-versa.

### Data analysis

A descriptive analysis for the overall mean, standard deviation (SD), median and range of ECOHIS scores and those for the individual domains were analyzed. For this initial exploratory analysis, the Kolmogorov-Smirnov test was used in order to check the normality distribution of the values. Then, analyses of covariance were carried out using the caries severity data as a covariant. Univariate Poisson Regression analysis with robust variance was performed to correlate the overall mean ECOHIS score to each clinical oral condition (types of TDI, AMT and ECC), gender and age. In this analysis, the outcome was employed as a count outcome, as performed previously [31,32], and rate ratios (RR) and 95% confidence intervals (95% CI) were calculated.

A multivariate model was later built with the covariates. These covariates were selected by a forward stepwise procedure. To enter the model, we considered variables with p < 0.20 and in order to be kept in the model, the variables should present p < 0.05. The severity of ECC adjusted the final multivariate model. For all analysis the statistical software STATA 8.0 (Stata Corp, College Station, USA) was used.

## Results

The children's mean (± SD) age was 3.8 years (± 1.11). From the 260 children, 137 (52.7%) were boys and 123 (47.3%) were girls.

AMT were present in 63 (24.2%) children, from which forty-four (16.9%) were children with anterior open bite and 19 (7.3%) were children with incisal overjet greater than or equal to 3 mm. From the 260 children, 87 (33.5%) had some type of TDI. Sixty-six (84.6%) were uncomplicated injuries and 21 (15.4%) were complicated injuries. Crown discoloration was present in 16 (6.1%) children. Ninety-four (36.2%) children were caries free, 87 (33.4%) were low severity and 79 (30.4%) were high severity.

The OHRQoL questionnaires were answered mostly by mothers (93.4%). Table 1 displays the mean, standard deviation, median and the range for the total ECOHIS score and for each domain. The mean overall score was 9.21. The items related to pain, irritation, difficulty in eating some foods, having trouble sleeping and difficulty in drinking hot or cold beverages were the most frequently reported on the child impacts section. Items related to the family being upset and feeling guilty were frequently reported on the family impacts section of the ECOHIS. Parents reported a more negative impact on the OHRQoL in relation to the child (69.3%) than the family (30.7%); 40.1% and 59.9% of the parents reported scores of 0 (floor effects) on the child's and family's sections, respectively. No ceiling effects were observed for either of the two sections. The maximum highest score was 30, reported on the child impact section, and 12, on the family impact section.

**Table 1:** Mean, standard deviation (SD), median and range observed in ECOHIS

ECOHIS	Mean ± (SD)	Median	Range observed
**ECOHIS total (0 - 52)**	9.21 ± 9.99	6	0 - 42
**Child impacts section**			
*Symptoms*	1.27 ± 1.41	1	0 - 4
*Function*	2.37 ± 3.17	1	0 - 14
*Psychological*	1.68 ± 2.30	0	0 - 8
*Self-image/social interaction*	0.69 ± 1.74	0	0 - 8
**Family impacts section**			
*Parental distress*	1.92 ± 2.39	0	0 - 8
*Family function*	0.75 ± 1.41	0	0 - 8

Overall, less than 3% of the sample responded "I don't know" to one or two items (results not shown). "I don't know" answers were most often observed for questions related to the difficulty in drinking hot or cold beverages and pronouncing some words. On the family impact, no "I don't know" responses were observed. No questionnaire was excluded from the analysis due to the "I don't know" responses.

Table 2 shows the mean difference between the types of TDI and AMT for each domain and for the overall ECOHIS. The presence of malocclusion did not show a negative impact on the overall OHRQoL score or in each domain. Complicated TDI showed a negative impact on the *symptoms *(p = 0.005), *psychological *(p = 0.029), *self image/social interaction *(p = 0.004) and *family function *(p = 0.018) domains of OHRQoL and in the overall mean ECOHIS score (p = 0.002).

**Table 2:** Mean difference between types of TDI and AMT for each domain and for overall ECOHIS

Oral clinical condition	n (%)	SYD	(±SD)	FD	(±SD)	PD	(±SD)	SSD	(±SD)	PDD	(±SD)	FFD	(±SD)	Mean ECOHIS Score	(±SD)
**Type of TDI**															
Absence	173 (66.5)	1.42^A^	1.46	2.45	3.33	1.75^A^	2.39	0.83^A^	1.92	1.97	2.47	0.79^A^	1.54	9.77^A^	10.64
Uncomplicated TDI	66 (25.4)	0.73^A^	1.17	1.79	2.53	1.18^A^	2.00	0.06^A^	0.39	1.48	1.98	0.44^A^	0.88	6.06^A^	6.90
Complicated TDI	21 (8.1)	1.86^B^	1.24	3.52	3.31	2.71^B^	2.10	1.48^B^	2.27	2.81	2.71	1.43^B^	1.43	14.48^B^	10.08
p - value		**0.005**†		0.084†		**0.029**†		**0.004**†		0.057†		**0.018**†		**0.002**†	
**AMT**															
Absence	197 (75.8)	1.35	1.45	2.38	3.20	1.75	2.39	0.76	1.83	1.97	2.48	0.77	1.42	9.54	10.34
Presence	63 (24.2)	1.06	1.27	2.35	3.11	1.46	1.98	0.46	1.42	1.75	2.08	0.70	1.39	8.19	8.84
p - value		0.168*		0.954*		0.383*		0.234*		0.481*		0.737*		0.315*	

SYD = Symptoms Domain FD = Function Domain PD = Psychological Domain SSD = Self-image/Social interaction Domain PDD = Parental Distress Domain FFD = Family Function Domain

* T-test

† covariance tests considering caries severity as covariable

Different letters (^A, B^) mean statistically different results (p < 0.05).

The univariate analysis shows that uncomplicated and complicated TDI and children having 3 or 4 years of age were correlated with the outcome variable (OHRQoL) (p < 0.05) (Table 3). The final multivariate model were adjusted with the severity of ECC and only the presence of complicated TDI showed an increased negative impact on the children's quality of life (RR = 1.90; 95% CI = 1.38 to 2.62; p < 0.001) (Table 4).

**Table 3:** Univariate analysis of association between the types of TDI and AMT on the overall ECOHIS

Univariated	n (%)	Robust RR (95% CI)	P - value
**Types of TDI**			
Absence	173 (66.5)	1.00	
Uncomplicated TDI	66 (25.4)	0.64 (0.46 - 0.87)	**0.005**
Complicated TDI	21 (8.1)	1.49 (1.06 - 2.07)	**0.020**
**AMT**			
Absence	197 (75.8)	1.00	
Presence	63 (24.2)	0.86 (0.63 - 1.16)	0.328
**ECC**			
Caries free (dmf-t = 0)	94 (36.2)	1.00	
Low severity (dmf-t = 1-5)	87 (33.4)	2.15 (1.49 - 3.11)	
High severity (dmf-t ≥ 6)	79 (30.4)	4.33 (3.06 - 6.14)	**< 0.001**
**Sex**			
Male	137 (52.7)	1.00	
Female	123 (47.3)	1.09 (0.84 - 1.42)	0.534
**Age**			
2 years	46 (17.7)	1.00	
3 years	60 (23.1)	1.71 (1.04 - 2.82)	**0.036**
4 years	66 (25.4)	2.05 (1.26 - 3.31)	**0.004**
5 years	88 (33.8)	1.54 (0.96 - 2.47)	0.073

**Table 4:** The multivariate fitted model of covariates associated to overall ECOHIS

Multivariated	Robust RR (95% CI)	P - value
**Types of TDI**		
Absence	1.00	
Uncomplicated TDI	0.89 (0.66 - 1.20)	0.441
Complicated TDI	1.90 (1.38 - 2.62)	**< 0.001**
**AMT**		
No	1.00	
Yes	0.97 (0.75 - 1.26)	0.821
**ECC**		
Caries free (dmf-t = 0)	1.00	
Low severity (dmf-t = 1-5)	2.02 (1.39 - 2.92)	**< 0.001**
High severity (dmf-t ≥ 6)	4.23 (2.96 - 6.05)	**< 0.001**
**Age**		
2 years	1.00	
3 years	1.12 (0.71 - 1.76)	0.618
4 years	1.13 (0.73 - 1.76)	0.580
5 years	1.11 (0.73 - 1.68)	0.622

## Discussion

This research evaluates the impact of TDI and AMT on the OHRQoL of preschool children, testing the Brazilian Portuguese version of the ECOHIS. We could observe that the occurrence of complicated TDI may cause a negative impact on OHRQoL of preschool children, whereas AMT does not. To the best of our knowledge, this is the first study which specifically evaluates the impact of TDI on OHRQoL in children with primary teeth.

The ECOHIS uses response options from parents to assess the frequency in which oral disease and treatment affect a child's OHRQoL. Child self-report is considered the standard for measuring perceived health-related quality of life; however, there are circumstances when parent proxy-report may be indicated (young age, presence of cognitive impairments, illness, or fatigue preventing self-report) [33]. Beyond that, there is evidence indicating that children younger than 6 years of age are unable to recall important details of events related to their health beyond 24 hours [34]; so practitioners must depend on parents when assessing a child's health status. A systematic review has concluded that valid information can be found out by the use of questionnaires when they are applied in adequate techniques. This information can be either provided by children or parents, even if they do not necessarily share similar opinions on OHRQoL. Although parents may report incomplete information about their children, possibly due to the lack of knowledge on some of their children's experiences, the children can still provide and complement the information given by parents [35]. The ECOHIS is one of the instruments which seems to have an appropriate assessment technique, so, it is possible to obtain valid and reliable information from preschool children concerning their OHRQoL [16,36].

Parental gender was found to be a predictor of the number of "I don't know" responses for oral symptoms, as fathers give such answers more than mothers [37]. In the present study, "I don't know" responses were most often observed when the father answered the questionnaire (results not shown), which may indicate that the fathers' poorer knowledge regarding the impact on OHRQoL of their children in relation to mothers. Beyond the parental gender, in a study comparing the level of agreement between parental and child (6-14 years) reports, Jokovic et al. 2004 [37] found that the child's age is a significant predictor of "I don't know" responses given by the parents in the oral symptoms, emotional well-being and social well-being items. This reflects the fact that as children get older they spend more time away from parental supervision and, therefore, they less experiences with parents [36]. In our study, less than 3% of the sample responded "I don't know" to one or two items, probably because preschool children need extra care and attention, and parents spend more time and have better knowledge about their children at this age.

The prevalence of 33.5% of TDI found in this study is in accordance with the literature [4-7], even though epidemiological studies include only visual assessment, which tends to underestimate the presence of TDI. As the present research did not use x-rays to assess TDI, there is some possibility that this prevalence is underestimated. Also concussions and subluxations are mild injuries which tend to solve themselves, but they may result in radiographic signs such as root resorption, pulp canal obliteration or periapical radiolucency (pulp necrosis). Also, root fractures are only found in radiographic exams.

The presence of complicated TDI was associated with a negative impact on OHRQoL in the overall mean ECOHIS score. This is probably due to symptoms frequently related to complicate TDI such as pain, irritation, difficulty in eating some foods, trouble sleeping and difficulty to drink hot or cold beverages. These were the most frequent ECOHIS responses reported on the child impacts section. Locker et al. [19] and Berger et al. [22] found similar results of negative impact on OHRQoL of schoolchildren when more severe levels of TDI were present.

Assessing each ECOHIS domain, complicated TDI showed a negative impact on the *symptoms*, *psychological, self image/social interaction *and *family function *domains of OHRQoL. The *symptoms *and *psychological *domains comprised items related to pain or discomfort that can lead to the child having trouble sleeping and/or being irritable because of dental problems or treatments. Probably, this pain related by the parents does not truly represent the pain the child feels at the moment of the TDI, but it could be due to sequelae from an untreated TDI, such as pulp inflammation or exposure, or excessive mobility of the tooth which suffered some kind of luxation. This situation could be prevented if parents were to look for urgent treatment soon after the TDI.

The negative impact of complicated TDI on the *family function *domain is probably because of the time this type of injury happens, there is always some urgency to deal with the problem, and therefore it results in parents missing work to take care of their child, or even spend extra time and money in dealing with dental care. This association was already reported by parents whose children had ECC [38,39].

The negative impact on the *self image/social interaction *domain caused by the complicated TDI, can be explained on the types of TDI that comprised this category. For example, dental avulsions can produce an aesthetic discomfort that sometimes is only solved when teeth are replaced. Also, lateral luxation, extrusive luxation and intrusive luxation change teeth position and suddenly damage the harmony of the smile. Vale et al [40] evaluated drawings of children aged 2 to 11 years, according to the Piaget's cognitive development scale, and found that children of all ages clearly represent their perception of what "beautiful teeth" and "ugly teeth" are. In such view, it may be expected that children who suffered severe TDI could avoid smiling and speaking.

Another issue is that ECOHIS evaluates the OHRQoL of the preschool children since birth. This is an advantage because it assesses the whole life instead of a short period of life [17]. However, according to Jabarifar et al. [17], there are two limitations when assessing the whole life: the period of assessment is different from child to child based on their age, and some parents can be confused whether they should include impacts of different periods. Regarding TDI, the time between the injury and the interview could influence the result since parents may not remember the occurrence of TDIs and their impact when the child was younger. Furthermore, recent, acute and painful TDI might cause more negative impact, since it is easier for parents to remember recent episodes which caused great discomfort to the child. To minimize this limitation, the interviewers were trained to explain that the child's whole life period should be taken into consideration when the parents answered the questions and all adverse oral conditions should be related in the interview.

The presence of AMT was not associated with a negative impact on OHRQoL in each domain or in the overall mean ECOHIS score. Foster Page et al. [41] and O'Brien et al. [42] described that the most significant impact of malocclusion on OHRQoL of children aged 11-14 years is psychosocial, affecting the emotional well-being and social domains. In preschool children, on the other hand, the results are different. One reason for this may be that the AMT evaluated in this research are often associated with non-nutritive sucking habits, such as the finger or pacifier sucking and prolonged use of bottle-feeding. So, many children at this age prefer the maintenance of these habits, leaving behind the oclusal and aesthetics changes produced by the bad position of teeth. Moreover, differently to severe TDI, where the change happens suddenly, in AMT, the changes in the dentition occur slowly and over the developmental stages of childhood and adolescence. Therefore, AMT usually goes by unnoticed to children and parents and most of them do not know the aesthetic, psychological and financial consequences that malocclusion can produce at more advanced ages.

Beyond that, analyzing the structure of the ECOHIS questionnaire, it can be observed that, despite the fact that the instrument has been validated to assess the impact of oral health problems in general, the questions are more suitable for assessing ECC and TDI rather than malocclusions. It seems that the ECOHIS was not developed specifically to measure the impact of different malocclusions on the OHRQoL. Also, some of the questions in the child *function *and *symptoms *domains are not necessarily relevant to children with malocclusion [30].

Differently to ECC, which has a strong association with socioeconomic factors, [43] some studies have showed a lack of association between TDI and such factors [5,13,44]. Others, however, showed a higher prevalence of TDI in an upper socioeconomic group [45,46]. Therefore, future studies on the impact of TDI on the quality of life should consider the socioeconomic conditions as well.

Considering that we selected patients who sought dental treatment, we only could extrapolate the results of this study to the dental office setting when children are taken prior to receiving dental treatment. Some studies have also assessed the impact of oral conditions on children's quality of life with convenience samples in hospitals or universities institutions [41,42,47,48]. Nevertheless, a limitation of the study is extrapolating the results to the general population. For that reason, future studies could be realized in order to assess the impact of TDI and AMT on a representative sample.

This study conclude that complicated traumatic dental injuries have a negative impact on the Oral Health Related Quality of Life of preschool children and their parents, but anterior malocclusions traits does not. Therefore, it is necessary to facilitate children's access to dental care services, especially when dealing with dental injuries, in order to avoid a later negative impact on their quality of life. Moreover, these results can help clinicians and researchers in their attempts to improve oral health outcomes for young children.

## Abbreviations

AMT: Anterior Malocclusion Traits; ECC: Early Childhood Caries; ECOHIS: Early Childhood Oral Health Impact Scale; CI: Confidence Intervals; OHRQoL: Oral Health-Related Quality of Life; RR: Rate Ratios; TDI: Traumatic Dental Injuries; WHO: World Health Organization criteria

## Competing interests

The authors declare that they have no competing interests.

## Authors' contributions

JMA was responsible for analysis and interpretation of data, helped the statistical analysis and drafted the manuscript; JA was responsible for the conception and design the study, acquisition, analysis and interpretation of data. TSC performed data acquisition, analysis and interpretation, helped the statistical analysis and draft the manuscript; FMM made statistical analysis and interpretation of data, and critical manuscript review; MTW performed analysis and interpretation of data, and critical manuscript review; MB was responsible for conception design and critical review; DPR was responsible for the conception and study design, and performed the final critical review. All authors read and approved the final manuscript.
